# Fracture Resistance of Simulated Immature Teeth Filled with Three Types of Calcium Silicate Cement after Intracanal Medication with Ca(OH)_2_: An Ex Vivo Study

**DOI:** 10.1155/2024/8386533

**Published:** 2024-04-26

**Authors:** Kuttalee Sriprasart, Suwit Wimonchit

**Affiliations:** ^1^Faculty of Dentistry, Srinakharinwirot University, Bangkok, Thailand; ^2^Department of Conservative Dentistry and Prosthodontics, Faculty of Dentistry, Srinakharinwirot University, Bangkok, Thailand

## Abstract

**Objectives:**

The aim of this study was to evaluate the 1-day fracture resistance of simulated immature teeth with an apical plug with ProRoot MTA, MTA Angelus, and RetroMTA after calcium hydroxide intracanal medication.

**Materials and Methods:**

Sixty extracted, single-rooted human mandibular premolars were randomly divided into six groups of 10 teeth each. Firstly, to standardize the 9 mm root length, the crowns were cut off at and 9 mm below the cementoenamel junction transversely. Simulations for immature apices were carried out by using No. 1–6 Peeso reamers to pass through the apex to obtain a diameter of 1.7 mm at the apical opening. One group served as a negative control without any treatment, while the other groups received 30 days of calcium hydroxide intracanal medication. Four groups were plugs with 4 mm of ProRoot MTA, MTA Angelus, RetroMTA, and gutta-percha, respectively. The last one was served as a positive control group without filling inside. After 1 day of incubation, specimens were vertically loaded in a universal testing machine at a crosshead speed of 1 mm/min until fracture occurred. The peak load to fracture (Newton) and fracture pattern were recorded. A one-way analysis of variance (ANOVA) followed by Tukey's HSD test was used for data analysis.

**Results:**

The highest mean load to fracture was shown in the negative control group (543.33 ± 37.17 N), followed by ProRoot MTA (432.82 ± 68.06 N), MTA Angelus (396.92 ± 59.93 N), RetroMTA (389.08 ± 56.25 N), and gutta-percha (283.28 ± 43.40 N), and the lowest belonged to the positive control group (239.98 ± 27.19 N). The significant differences were found between both the control and experimental groups with an apical plug with calcium silicate cement (*p*  < 0.05). There were no significant differences among those three apical plugs (*p*  > 0.05).

**Conclusion:**

Apical plugs with ProRoot MTA, MTA Angelus, and RetroMTA had an immediate strengthening effect on simulated immature teeth after calcium hydroxide intracanal medication had been used.

## 1. Introduction

Cessation of root development due to traumatic injuries or pulp necrosis in children results in a large root canal, a thin, fragile root canal wall, and an open apex [1]. These situations cause difficulty in instrumentation, prevent apical seal formation, and easily break teeth during function [2]. To solve these problems, several techniques are proposed to both disinfect and reinforce the root canal wall [3].

The use of calcium hydroxide is one of the methods to disinfect the root canal system. Its alkalinity eliminates both the bacteria and necrotic tissue and promotes the apical hard tissue barrier [4]. The duration of dressing with calcium hydroxide varies according to preoperative situations. For the treatment of infected root canals, a period of 1–4 weeks is generally stated [5]. Fuss et al. [6] found that, after 30 days of calcium hydroxide exposure, it was still able to maintain a bactericidal pH level within the root canal, which was essential for the denaturing and detoxification of bacterial products such as lipopolysaccharide [7]. However, this material has a lot of disadvantages, such as tissue irritation, long-term hard tissue barrier activation, and a fracture strength reduction of root canal wall [8]. Dentin reaction from its high alkalinity and the deterioration of linkage between hydroxyapatite and collagen fibril may be the explanation of this event [9–11]. Sahebi et al. [12] demonstrated that calcium hydroxide intracanal placement for 30 days resulted in about a 15% dentin strength reduction.

Immature teeth are more responsive to occlusal forces or subsequent trauma than normal. Cvek [13] demonstrated that immature teeth, particularly the cervical third of the crown, were more prone to fracture. It occurred during chewing or biting (83%), as well as a minor injury (17%). Due to weak immature roots, it is advantageous to reinforce them, especially immediately after obturation, to decrease their fracture chance.

Apical plugs or intraradicular reinforcement are major root strengthening methods for immature roots after canal cleansing [14]. The material used for reinforcement must meet the following criteria: be easy to use in clinic, be able to bond to dentin, and provide an effective barrier against microleakage [15]. Calcium silicate cement has been suggested by Camilleri [16] as a viable choice for apical plugs due to its good sealing ability, antibacterial properties, and biocompatibility, as well as fracture resistance improvement [17]. Mineral trioxide aggregate (MTA) was recommended for its strengthening effect by Mente et al. [18]. New calcium silicate cements, such as MTA Angelus and RetroMTA, have recently been reported to be in clinical use. These materials have mechanical and physical properties similar to MTA [19, 20]. According to Ancheun and Wimonchit [21], immature teeth that are entirely filled with ProRoot MTA, MTA Angelus, and RetroMTA can increase fracture resistance.

The weakening effect of intracanal medication with calcium hydroxide was proven, which can deteriorate the strength of immature teeth and result in tooth breakage at any function time [9–12]. If an immediate root strengthening effect can be obtained, it should reduce that chance. Considering this problem, the aim of this study was to compare the 1-day fracture resistance of simulated immature teeth with an apical plug with ProRoot MTA, MTA Angelus, and RetroMTA after calcium hydroxide intracanal medication. The null hypothesis was that apical plugs with ProRoot MTA, MTA Angelus, and RetroMTA have no different strengthening effects on the fracture resistance of simulated immature teeth after calcium hydroxide intracanal medication has been used.

## 2. Materials and Methods

### 2.1. Tooth Selection

The study protocol was submitted to the ethics committee of Srinakharinwirot University and its approval was obtained (SWUEC/X-302/2565). The study was conducted in accordance with the principles of the Declaration of Helsinki. A G ^*∗*^Power calculation (software version 3.1.9.4) was performed to estimate the sample size using an effect size of 0.70 with a power of 0.80 to determine a minimum sample size of 10 in each group. The statistical significance level was set at 95% (*p* = 0.05).

Sixty extracted single-rooted human mandibular premolars were selected and stored in 0.1% thymol solution. All of them were examined for free of root caries, no crack or fracture, and external root resorption under a dental operating microscope (Carl Zeiss OPMI Proergo; Carl Zeiss Meditec AG, Jena, Germany) at 16x magnification. Digital periapical radiographs (FOCUS™ Intraoral X-ray, KaVo, Finland) were taken in both the buccolingual and mesiodistal directions to ensure no calcification, no internal root resorption, and a straight single root canal. The buccolingual and mesiodistal dimensions of the root were measured using a digital vernier caliper (Mitutoyo, Tokyo, Japan) at the cementoenamel junction (CEJ). Teeth with similar buccolingual and mesiodistal dimensions were selected (7.21 ± 0.46 and 5.07 ± 0.27 mm, respectively).

### 2.2. Tooth Preparation

All of them were decoronated at CEJ, and the root ends were cut off, leaving a 9 mm root length [22], followed by pulp removal with a barbed broach (Kerr Corporation, Orange, CA). In order to create immature roots with an apical diameter of 1.7 mm, the root canals were instrumented with Peeso reamers (size 1–6, Mani lnc., Tochigi, Japan) until a size 6 Peeso (1.7 mm) could be passed through the apex [17]. The roots were irrigated with 2 ml of 2.5% sodium hypochlorite (NaOCl) (M Dent, Bangkok, Thailand), followed by 2 ml of 17% ethyldiaminetetraacetic acid (EDTA) (M Dent, Bangkok, Thailand), finally rinsed with 2 ml of distilled water after instrumentation, and dried with paper points. After tooth preparation, the teeth were reexamined under a dental operating microscope at 16x magnification to exclude any cracks or fractures caused by this procedure. A digital vernier caliper was used to measure the remaining dentin thickness at CEJ level [23]; therefore, only teeth with similar remaining dentin thickness were included (2.03 ± 0.09 mm). All roots were randomly divided into six groups (*n* = 10 each): group 1 negative group; group 2 positive group; group 3 ProRoot MTA group; group 4 MTA Angelus; group 5 RetroMTA group; and group 6 gutta-percha group.

### 2.3. Treatment Procedures

The roots in group 1 received no treatment. The other roots were dressed with calcium hydroxide intracanal medication (Ultracal® XS; Ultradent Product, Inc.), sealed with temporary restoration (Cavit-G; 3M ESPE, St. Paul, Minnesota, USA), and then incubated at 37°C with 100% humidity for 30 days. After that time, the intracanal medication was removed with a size 80 stainless steel K-file (Dentsply Sirona, Ballaigues, Switzerland) and then rinsed with 2 ml of 2.5% NaOCl and 2 ml of 17% EDTA, followed by irrigation with 2 ml of distilled water. The canals were dried with paper points. The roots in group 2 were left unfilled, while the other 40 roots received ProRoot MTA, MTA Angelus, RetroMTA, and gutta-percha as assigned.

In group 3, in order to create a 4-mm-thick MTA plug, ProRoot MTA (ProRoot® MTA, Dentsply Tulsa Dental, Tulsa, OK, USA) was mixed according to the manufacturer's recommendation and packed into the simulated open apex root canal with an endodontic plugger (B&L Condenser; B&L Biotech Inc., Fairtax, VA, USA) under ultrasonic vibration (P5 Newtron XS; Acteon, Saint Neots, UK, and Ultrasonic Scaling tip, Satelec®) [24].

In groups 4 and 5, MTA Angelus (MTA Angelus®, Angelus, Londrina, PR, Brazil) and RetroMTA (RetroMTA®, BioMTA, Seoul, Korea) were used in the same manner in group 3 with the recommended manufacture ratio, respectively [24].

In group 6, AH Plus sealer (Dentsply DeTrey GmbH, Konstanz, Germany) was mixed and applied to the root canal wall with K-file size 80, followed by thermoplasticized gutta-percha compaction (Obtura Spartan Endodontics, Algonquin, IL) [24].

**Figure 1:**
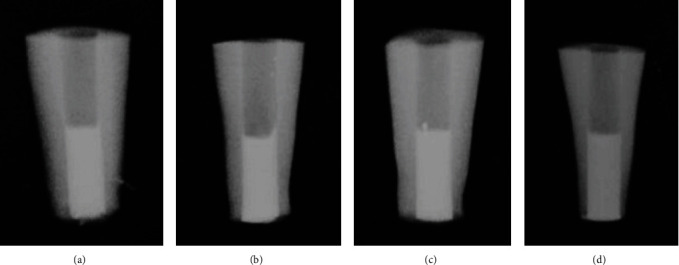
Radiographic images of (a) group 3: ProRoot MTA, (b) group 4: MTA Angelus, (c) group 5: RetroMTA, and (d) group 6: gutta-percha.

The homogeneity of these materials was confirmed with two dimensions of digital periapical radiographs (Figure 1). All roots were sealed with temporary restoration and then incubated at 37°C and 100% humidity for 1 day.

### 2.4. Fracture Testing

Before the fracture testing, the temporary restoration was removed from the coronal section of each specimen. The root surfaces were marked 2 mm below the CEJ and put down in molten wax (modeling wax; Associated Dental Products Ltd., Swindon, UK) for 1 s to form a 0.2–0.3-mm layer of wax [22]. The wax-dipped roots were submerged in plastic cylindrical molds filled with self-curing acrylic resin (GC America Inc., Illinois, USA). The roots were removed from the molds before the completely set acrylic resin and wax were removed by rinsing with warm water. The molds were dried and filled with silicone rubber material (Silagum®; light body impression, DMG, Germany). Immediately afterward, the roots were reinserted into the molds until there was a 2-mm gap between the coronal portion of the root and the top of the acrylic resin, and the excess silicone rubber material was removed with a No.11 scalpel blade (Feather, Osaka, Japan) [24].

**Figure 2:**
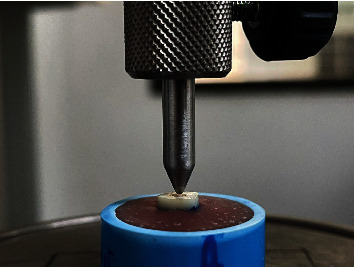
A vertical compression force was delivered to the root using a universal testing machine.

Specimens were vertically compressively loaded in a universal testing machine (EZTest; Shimadzu, Kyoto, Japan). With a cross-head speed of 1 mm/min, the 1 mm diameter of conical shaped indenter could slightly move down into the center of the coronal end and make contact with the radicular dentin until fracture occurred (Figure 2) [25]. The maximal force (Newtons) and fracture pattern were also recorded as supraacrylic resin, subacrylic resin, and vertical root fracture [24].

### 2.5. Statistical Analysis

Statistical analysis was performed with SPSS (Statistics 27; SPSS Inc., Illinois, USA). A one-way analysis of variance (ANOVA) was used to evaluate the difference among the buccolingual and mesiodistal dimensions and the root dentin thickness at the CEJ level of the roots. Following the fracture test, the mean load to fracture and the standard deviation for each group were estimated. The data were analyzed by one-way ANOVA. A Tukey's HSD test was used to determine any significant differences between groups at a 95% confidence interval.

## 3. Results

From tooth dimensions and root dentin thickness measurements, both before and after root canal preparation, no significant difference was found among the six groups (*p*  > 0.05) indicating that the tooth size similarity of each group could be obtained (Tables 1 and 2).

**Figure 3:**
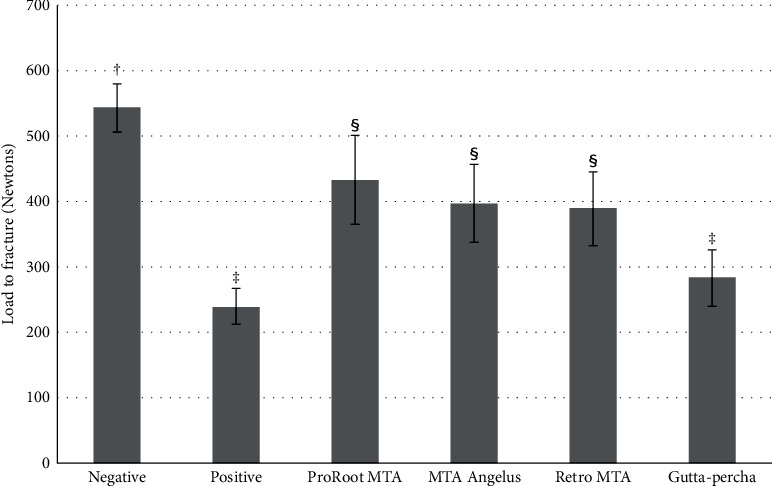
A graphic representation of the mean load to fracture of the six tested groups. Different alphabets indicated significant differences between groups (*p*  < 0.05).

**Figure 4:**
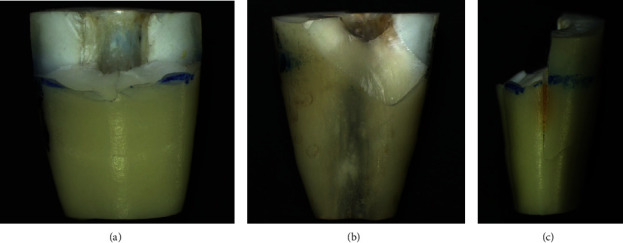
Fracture patterns: (a) supraacrylic resin fracture, (b) subacrylic resin fracture, and (c) vertical root fracture.

The mean immediate 1-day load to fracture and standard deviation are presented in Figure 3 and Table 3. The highest load to fracture (543.33 ± 37.17 N) was found in group 1, while the lowest (239.98 ± 27.19 N) was detected in group 2. The use of materials plugged into simulated root canals was found to give more fracture resistance than positive control group (group 2). A one-way ANOVA revealed that at least two of the groups statistically differed from the others. Tukey's HSD test did not show significant differences (*p*  > 0.05) between the positive control group (group 2) and group 6 and among the experimental groups. Significant differences (*p*  < 0.05) were found between the positive control group (group 2) and the experimental groups (groups 3, 4, and 5). Three fracture patterns were detected (Figure 4). The most common fracture pattern was the subacrylic resin fracture (Table 4).

## 4. Discussion

In this study, a human immature teeth simulation model were used to investigate the effect of calcium hydroxide intracanal medication, which confirmed the reduction of fracture strength in agreement with Sahebi et al. [12]; however, the rate of fracture strength reduction in the present report seemed to be higher than in the other. This present result suggested that about 50% reduction in fracture strength can be obtained after 30 days of that medication. These differences may be due to the source of the tooth specimen tested, different open apex simulation methods, and the intensity of calcium hydroxide. Andreasen et al. [8] proposed that up to a 50% reduction in fracture strength should be presented in cases of intracanal medication for more than 3 months in bovine teeth. Animal teeth are structurally similar to but morphologically different from human teeth; specifically, they have thicker dentinal walls as well as a larger root canal [26]. For that reason, only minimal strength depletion can be detected [8]. These were different from the study in human immature teeth, which found more strength reduction [8, 27].

The change in linkage between hydroxyapatite crystals and collagenous networks caused by the alkalinity of calcium hydroxide should be an explanation for the reduction in fracture strength. The hydroxyl ion may neutralize the acidic organic components that contain phosphate and carboxylate groups, which act as bonding agents, resulting in a breakdown of some bonding areas and, ultimately, the weakening of the simulated root.

The other reason for the weakening effect may be the minimal tooth structure of the simulated specimen in this study. Fuss et al. [6] showed, in the case of a mature tooth, that the hydroxyl group could penetrate through the dentin wall and be detected at the periphery when calcium hydroxide was placed inside the root canal. Therefore, its alkalinity easily diffused all over the root dentin in this experiment, and the dissolving effect of dentin could be seen 30 days after placement.

When plugging materials in the root canal, the mean value of fracture resistance seems to increase for every material used. Nevertheless, the use of gutta-percha with AH Plus did not change the significant load to fracture value from group 2, as shown in Figure 3. It may be due to the low bonding value of AH Plus between the dentin and gutta-percha [28], as shown in the finding of Bortoluzzi et al. [29]. Moreover, the poor cohesive bond strength and lower modulus of elasticity of gutta-percha (0.074–0.079 GPa) as compared to dentin (14–18.6 GPa) [30] may be another explanation for an earlier fracture than other plugging materials.

The strengthening effect of calcium silicate cement, both from sintering manufacturing technique like ProRoot MTA and MTA Angelus and synthetic manufacturing technique like RetroMTA, was proved in this experiment. This is consistent with the findings of Sarraf et al. [31], who reported that root canal space filling with ProRoot MTA contributed to higher fracture resistance. These results showed that apical plugs using ProRoot MTA, MTA Angelus, and RetroMTA immediately regain significant root fracture resistance by almost 60%–80% of their original strength as well as the report of Andreasen et al. [32], who showed that root filling with MTA after 30 days of calcium hydroxide intracanal treatment increased tooth fracture resistance. Because of the physicochemical interaction between MTA-based materials and root canal walls, it was determined that MTA is a bioactive material [33]. A hydroxyapatite layer was formed on the surface of the material and appeared to create a chemical bond with dentin via a diffusion-controlled reaction between its apatite surface and dentin. This reaction enhanced the pushout bond strength, sealability, and fracture resistance [34].

According to a finite element analysis study [35], a material with a similar modulus of elasticity to dentin may reinforce weakening roots and minimize dentin stress during load. The modulus of elasticity of calcium silicate cement is around 15–30 GPa, which is close to the modulus of elasticity of dentin (14–18.6 GPa) [36]. When they were placed in the root canal. Our findings revealed that all of the materials tested significantly reinforced the immature roots. However, no significant differences were observed among the three materials, which may be attributed to the fact that dicalcium and tricalcium silicate are the major compositions of these materials. In addition to the approximate mechanical properties of ProRoot MTA, MTA Angelus, and RetroMTA, the null hypothesis is accepted.

In the current study, all teeth were standardized to the same length and width to control the factors that can affect the strength of the root. Additionally, a standardized tooth preparation and treatment process was adopted to control any confounding variables [26]. The teeth were decoronated at the CEJ level to avoid the potential impacts of cervical defects on the testing results and to focus the loading on the roots rather than the entire teeth. Following that, the canals were resected at the root end and instrumented with Peeso reamers beyond the apex to mimic the immature roots of stage 3 root development of the Cvek classification [17]. Stuart et al. [15] reported that canal wall reinforcement of teeth with a canal diameter of 1.5 mm or less may not be necessary; therefore, the canals were enlarged to a diameter of 1.7 mm.

For fracture testing, acrylic resin was utilized as the embedding material in our study because it has been shown to be capable of imitating the bones' ability to sustain compressive stresses during mastication [25], while the periodontal ligament-like structure is crucial in distributing stress in this model [37]. The PDL simulation procedure uses wax that has a melting point of about 60°C. Hayashi et al. [38] reported that heat temperatures lower than 200°C do not decrease the mechanical properties of human dentin; therefore, they do not affect the strength of teeth. Light body condensation silicone impression was proven to imitate periodontal ligament because, according to Jamani et al. [39] report, its modulus of elasticity was 0.31–0.35 MPa, which is near the elasticity of human periodontal ligament (0.12–0.96 MPa) [40]. Furthermore, in our study, the roots were embedded in acrylic, leaving a 2-mm gap between the top of the acrylic and the root surface at the CEJ level, which resembles the physiologic space found clinically between the alveolar bone crest and the CEJ [41].

Various studies have used different methods for transmitting force to the teeth to simulate the force that leads to fracture. Force was applied in the labiolingual [17], linguolabial [15], and vertical directions [25] in these studies. Considering Soliman et al. [42] stated that mandibular posterior teeth sustain vertical forces more than lateral forces during root canal obturation and occlusion, vertical forces were applied to the roots in the current study.

Analysis of the most common fracture pattern of the studied specimen showed subacrylic fractures, which represent the fractures below the gingival attachment. This pattern was in agreement with the findings of Karapinar-Kazandag et al. [22]. In clinical situation, they came from both iatrogenic and noniatrogenic factors that could propagate cracks and end up in fractures [43].

The limitations of this study may include the period of time and the incorporation of a single load in the fracture test. To mimic intraoral conditions, further studies should be conducted with thermocycling and dynamic fatigue loading. From this finding, it was considered that calcium silicate cements should be the choice of material used in immature teeth. They should reinforce the weak, incompletely root-formed teeth with both immediate and late reactions. The different mechanisms of those reactions should have been proven in the future.

## 5. Conclusion

Apical plugs with ProRoot MTA, MTA Angelus, and RetroMTA had an immediate strengthening effect on simulated immature teeth after calcium hydroxide intracanal medication had been used.

## Figures and Tables

**Table 1:** The buccolingual and mesiodistal dimensions (in millimeters) of the root at the cementoenamel junction (CEJ).

Groups	Mean ± SD	
	Buccolingual	Mesiodistal
Negative control	7.16 ± 0.26	5.01 ± 0.23
Positive control	7.16 ± 0.43	5.03 ± 0.33
ProRoot MTA	7.16 ± 0.34	5.17 ± 0.27
MTA Angelus	7.02 ± 0.44	5.08 ± 0.19
RetroMTA	7.42 ± 0.68	5.12 ± 0.29
Gutta-percha	7.38 ± 0.50	5.08 ± 0.35
*p* Value	0.37	0.77

**Table 2:** The root dentin thickness (in millimeters) at the cementoenamel junction (CEJ) after tooth preparation.

Groups	Mean ± SD
	Root dentin thickness
Negative control	2.03 ± 0.06
Positive control	2.03 ± 0.10
ProRoot MTA	2.01 ± 0.07
MTA Angelus	2.05 ± 0.10
RetroMTA	2.03 ± 0.07
Gutta-percha	2.02 ± 0.13
*p* Value	0.97

**Table 3:** The mean load to fracture (in Newtons) and standard deviation (SD) of the six tested groups.

Groups	Mean ± SD	Minimum	Maximum
Negative control	543.33 ± 37.17^†^	506.55	626.50
Positive control	239.98 ± 27.19^‡^	200.28	277.08
ProRoot MTA	432.82 ± 68.06^§^	325.75	541.53
MTA Angelus	396.92 ± 59.93^§^	328.55	507.88
RetroMTA	389.08 ± 56.25^§^	314.95	477.54
Gutta-percha	283.28 ± 43.40^‡^	236.65	363.90

Different alphabets indicated significant differences between groups (*p*  < 0.05).

**Table 4:** Fracture pattern in the number and percentage of six groups.

Groups	Fracture pattern					
	Supraacrylic resin		Subacrylic resin		Vertical root fracture	
	*n*	(%)	*n*	(%)	*n*	(%)
Negative control	5	50	5	50	0	0
Positive control	3	30	5	50	2	20
ProRoot MTA	6	60	3	30	1	10
MTA Angelus	5	50	4	40	1	10
RetroMTA	4	49	5	50	1	10
Gutta-percha	4	49	4	40	2	20
Total	26	43.3	27	45	7	11.7

## Data Availability

The authors confirm that the data supporting the findings of this study are available within the article.
